# Supplementary material for: METTL3 Promotes the Differentiation of Goat Skeletal Muscle Satellite Cells by Regulating MEF2C mRNA Stability in a m6A-Dependent Manner
Source: Int J Mol Sci. 2023 Sep 14;24(18):14115. doi: 10.3390/ijms241814115 (PMC10531580; doi:10.3390/ijms241814115)
Supplement: Supplementary file 1 [file ijms-24-14115-s001.zip › Supplementary Material.pdf]

Supplementary Information

METTL3 promotes the differentiation of goat skeletal muscle satellite cells by regulating MEF2C mRNA stability in m^{6}A-dependent manner

Sen Zhao [1,2†], Jiaxue Cao [1†], Yanjin Sun [1,2], Helin Zhou [1,2], Qi Zhu [1,2], Dinghui Dai [1], Siyuan Zhan [1,2], Jiazhong Guo [1,2], Tao Zhong [1,2], Linjie Wang [1,2], Tianzeng Song [3], Li Li [1,*] and **Hongping Zhang [1,2,*]**

1 Farm Animal Genetic Resources Exploration and Innovation Key Laboratory of Sichuan Province, Sichuan Agricultural University, Chengdu 611130, Sichuan, China;

2 Key Laboratory of Livestock and Poultry Multi-omics, Ministry of Agriculture and Rural Affairs, College of Animal and Technology, Sichuan Agricultural University, Chengdu 611130, Sichuan, China;

3 Institute of Animal Science, Tibet Academy of Agricultural and Animal Husbandry Sciences, Lhasa 850009, China;

zhaosen97@126.com (S.Z.); jiaxuecao@sicau.edu.cn (J.C.); s18098064595@163.com (Y.S.); a152136157@163.com (H.Z.); 15296542810@139.com (Q.Z.); 71317@sicau.edu.cn (D.D.); siyuanzhan@sicau.edu.cn (S.Z.); jiazhong.guo@sicau.edu.cn (J.G.); zhongtao@sicau.edu.cn (T.Z.); wanglinjie@sicau.edu.cn (L.W.); songtianzeng123@sina.com (T.S.);

† These authors have contributed equally to this work and share first authorship.

* Correspondence: lily@sicau.edu.cn (L.L.) and zhp@sicau.edu.cn (H.Z.)

Supplementary Figure

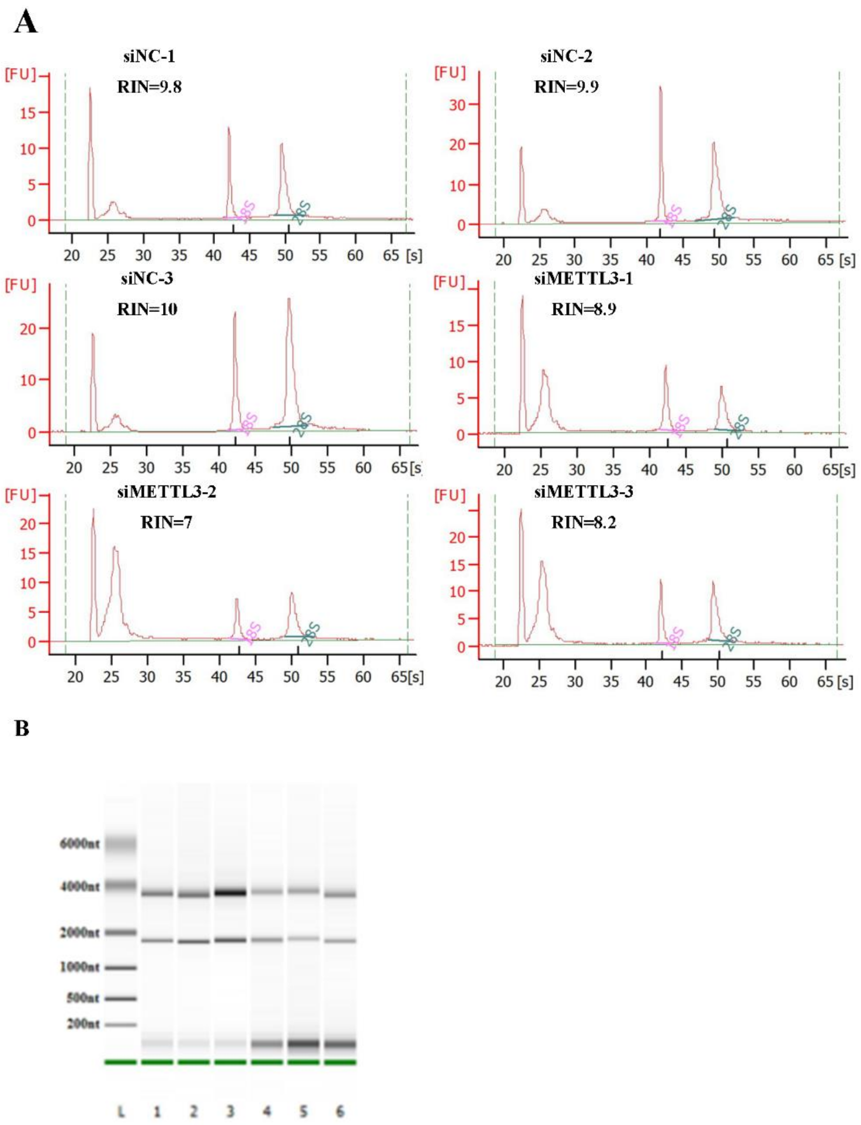

Figure S1. RNA quality detection for mRNA-Seq. (A) RNA quality was analyzed using an Agilent 2100 bioanalyzer, and RIN values were used to assess integrity. (B) Gel electrophoresis of total RNA.

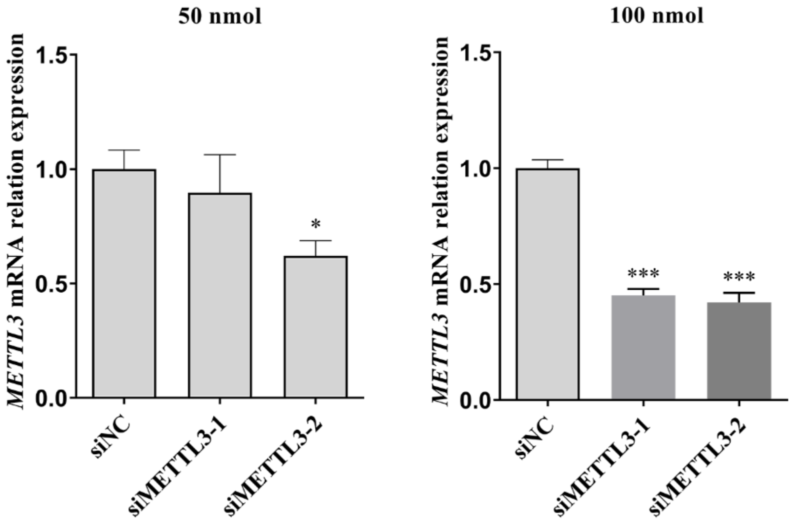

Figure S2. Determine the optimal transfection concentration of siMETTL3. The knockdown efficiency of different siMETTL3 was verified by qPCR analysis in MuSCs. Mean values ± SEM, *$p < 0.05$, ***$p < 0.001$.

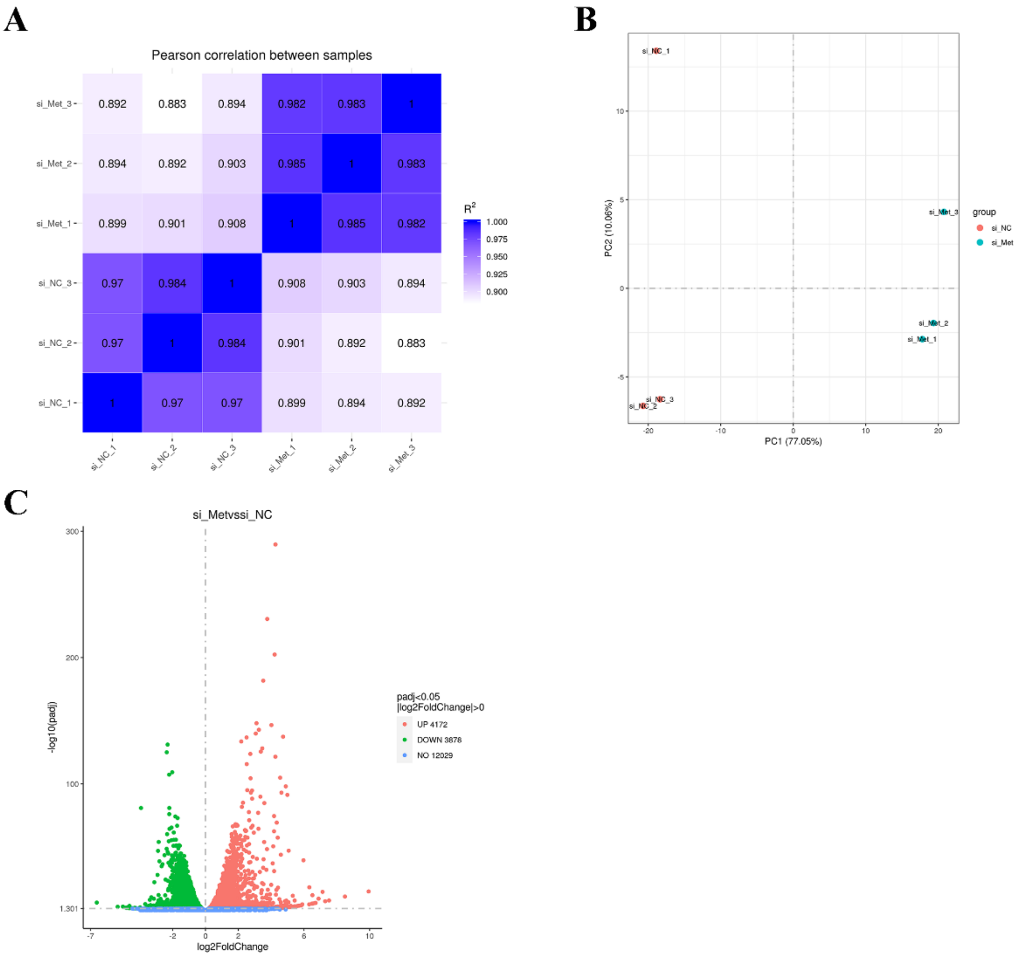

Figure S3. Identification of DEGs between siNC and siMETTL3-transfected cells. (A) Heat map of correlation (B) Results of principal component analysis. (C) Volcano map of DEGs.

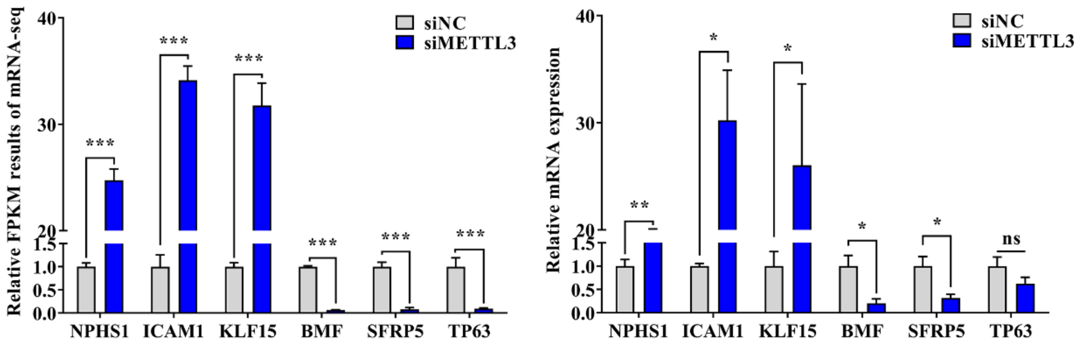

Figure S4. Verify mRNA-seq results. qPCR was used to validate some randomly selected DEGs (up and down-regulated) from mRNA-seq. Mean values ± SEM, *$p < 0.05$, **$p < 0.01$, ***$p < 0.001$, ns, no significance.

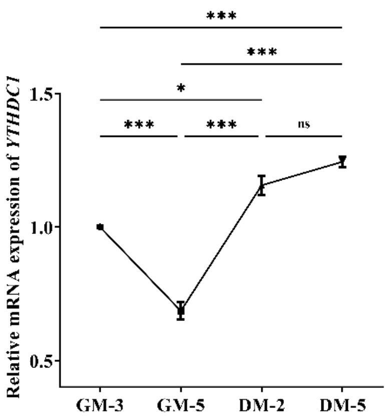

Figure S5. Expression patterns of MEF2C in MuSCs. qPCR analysis of MEF2C expression during the GM and DM of MuSCs. Mean values ± SEM, **p* < 0.05, ****p* < 0.001, ns, no significance.

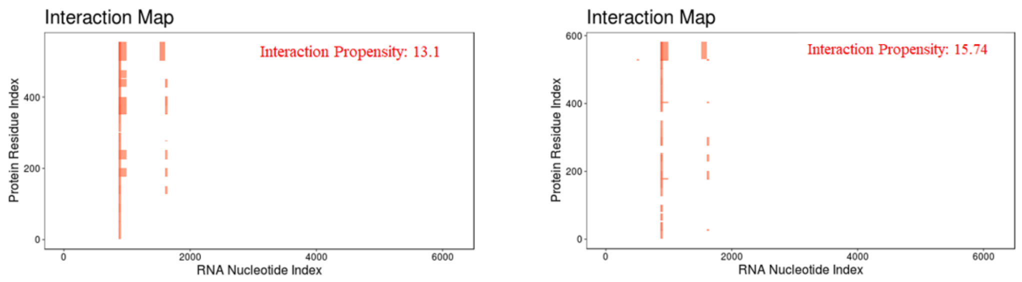

YTHDF1-*MEF2C* mRNA **YTHDF2-*MEF2C* mRNA**

Figure S6. catRAPID Omics v2.0 predicted the binding potential of YTHDF1 and DF2 to *MEF2C* mRNA. Interaction Propensity represents the probability of protein and RNA interaction.

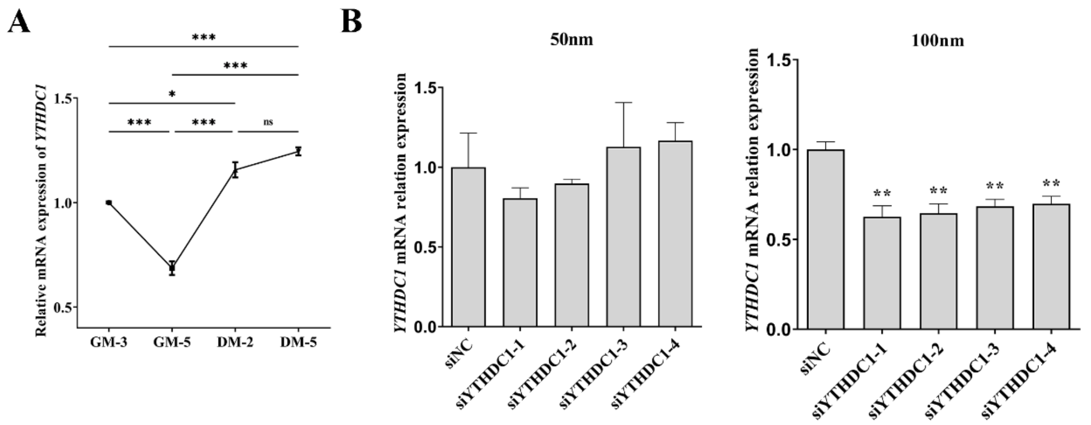

Figure S7. The expression of YTHDC1 and the optimal transfection concentration of siYTHDC1 were determined in MuSCs. (A) qPCR analysis of YTHDC1 expression during the GM and DM of MuSCs. The knockdown efficiency of different siYTHDC1 was verified by qPCR analysis in MuSCs. Mean values ± SEM, **p* < 0.05, ***p* < 0.01, ****p* < 0.001, ns, no significance.

Table S1. Details for siRNA sequence.

siRNA Name	Sequence
siMETTL3	CCCGGTTCAAGCAAAGATA
siYTHDC1-1	CGAGATAGAGGACGTGATA
siYTHDC1-2	CGTGATAGAGAAAGAGAAA
siYTHDC1-3	CCACATGAAGCAAGATACA

Table S2. Sequences of primers.

Target genes	Forward primer (5′-3′)	Reverse primer (5′-3′)
CDS cloning		
MEF2C-CDS	ATGGGGAGAAAAAAGATTCAGATTA	TGTTGCCCATCCTTCAGAGAG
HR-MEF2C	Xho1-F: CTACCGGACTCAGATCTCGAGATGGGGAGAAAAAAGATTCAGATTA	HindIII-R: CGACTGCAGAATTCGAAGCTTTGTTGCCCATCCTTCAGAGAG
RT-qPCR		
METTL3	TGTGCAACCCAACTGGATCA	ATCTTTGCTTGAACCGGGCA
MEF2C	ATCCTGA TGCAGACGATTCAG	GGTGGAACAGCACACAATCTT
GAPDH	GCAAGTTCCACGGCACAG	GGTTCACGCCCATCACAA
MyoD	GTGCAAACGCAAGACGACTA	GCTGGTTTGGGTTGCTAGAC
MyoG	GGACCCTACAGATGCCCACA	TTGGTATGGTTTCATCTGGG
MyHC	CCACATCTTCTCCATCTCTG	GGTTCCTCCTTCTTCTTCTC
MEF2C-1728	ATTGGACTCACCAGACCTT	TCATGTTGCCCATCCTTC
YTHDC1	TGGACGTGATGGACAGGA	TTGATCGGGGCTGAGAATGC

Table S3. Information of dual-luciferase primers.

Target genes	Forward primer (5′-3′)	Reverse primer (5′-3′)
HR-MEF2C-1728	Xho1-F: AATTCTAGGCGATCGCTCGAGATTGGACTCACCAGACCTTCGC	Not1-R: ATTTTATTGCGGCCAGCGGCCGCTCATGTTGCCCATCCTTCAGA

Table S4. Quality summary of mRNA-seq data.

Sample	raw_reads	clean_reads	clean_bases	Q20	Q30	GC_pct
si_NC_1	45303764	40764966	6.11G	97.75	93.89	52.56
si_NC_2	45943492	43164328	6.47G	97.75	93.73	51.36
si_NC_3	41434124	39277494	5.89G	97.86	93.92	51.33
si_Met_1	42041002	38468530	5.77G	97.75	93.7	49
si_Met_2	44244506	40880020	6.13G	97.12	92.39	48.61
si_Met_3	42134598	38663824	5.8G	97.96	94.09	48.62

Table S5. The genomic mapping results of clean reads.

Sample	total_reads	total_map(%)	unique_map(%)	multi_map(%)
si_NC_1	40764966	39546334 (97.01%)	37229398 (91.33%)	2316936 (5.68%)
si_NC_2	43164328	41942797 (97.17%)	39230233 (90.89%)	2712564 (6.28%)
si_NC_3	39277494	38108689 (97.02%)	33813297 (86.09%)	4295392 (10.94%)
si_Met_1	38468530	37384668 (97.18%)	35831944 (93.15%)	1552724 (4.04%)
si_Met_2	40880020	39446827 (96.49%)	37678609 (92.17%)	1768218 (4.33%)
si_Met_3	38663824	37689485 (97.48%)	36025393 (93.18%)	1664092 (4.3%)
